# The deep vein thrombosis of lower limb after total hip arthroplasty: what should we care

**DOI:** 10.1186/s12891-021-04417-z

**Published:** 2021-06-15

**Authors:** Xinyan Yu, Yingying Wu, Rende Ning

**Affiliations:** 1 Department of nursing, The Third Affiliated Hospital of Anhui Medical University, No. 390 Huaihe Road, 230061 Hefei City, Anhui Province China; 2 Department of nursing, Shannan people’s Hospital, Sare Road, Naidong District, Shannan City, 856011 Tibet Autonomous Region China

**Keywords:** deep vein thrombosis, total hip arthroplasty, management, nursing, care

## Abstract

**Background:**

Deep vein thrombosis (DVT) of lower limb is one of the common complications after total hip arthroplasty(THA), we aimed to evaluate the potential risk factors of DVT of lower limb in patients with THA, to provide insights into the management of THA.

**Methods:**

Patients who underwent THA in our hospital from January 1, 2017 to November 30, 2020 were included. The personal characteristics and clinical data of DVT and no-DVT patients were compared and analyzed. Logistic regression analyses were perfomed to identify the potential risk factors of DVT in patients with THA.

**Results:**

A total of 182 THA patients were included, the incidence of DVT of lower limb in patients with THA was 19.78 %. There were significant differences in the age, BMI, diabetes, number of replacement, duration of surgery, type of prosthesis and duration of days in bed between DVT and no-DVT patients(all *P* < 0.05). And there were no significant differences in the gender, hypertension, hyperlipidemia, preoperative D-dimer, type of anesthesia and anticoagulant drugs use(all *P* > 0.05). Logistic regression analysis indicated that age > 70y(OR4.406, 95 %CI1.744 ~ 6.134), BMI ≥ 28(OR2.275, 95 %CI1.181 ~ 4.531), diabetes(OR3.949, 95 %CI1.284 ~ 5.279), bilateral joint replacements(OR2.272, 95 %CI1.402 ~ 4.423), duration of surgery ≥ 120 min(OR3.081, 95 %CI1.293 ~ 5.308), cemented prosthesis(OR2.435, 95 %CI1.104 ~ 4.315), and duration of days in bed > 3 days(OR1.566, 95 %CI1.182 ~ 1.994) were the risk factors of DVT of lower limb in patients with THA.

**Conclusions:**

DVT in the lower limb after THA is common, and its onset is affected by many factors. In clinical work, attention should be paid to identify the risk factors for DVT and targeted interventions are highlighted to prevent the postoperative DVT.

## Background

Total hip arthroplasty (THA) is a common surgical treatment in the department of orthopedics and one of the effective treatments for end-stage hip joint diseases, mainly for the elderly[1]. THA is mainly used to treat joint pain and dysfunction caused by hip joint disease, including hip joint osteoarthritis, femoral head necrosis, bone neck fractures and so on[2]. At present, more than 500,000 people worldwide receive artificial joint replacement due to fractures, osteoarthritis, bone tumors and other diseases each year[3]. In China, 30,000 to 50,000 people undergo THA every year[4, 5]. Through artificial hip replacement, it can relieve joint pain, improve joint function, and correct deformity. And with proper postoperative functional exercise, the patient’s hip joint function may meet the needs of daily life and improve the quality of life[6].

Deep venous thrombosis (DVT) is a common complication after hip arthroplasty, with an incidence rate of 40–60 %[7]. The formation of DVT of the lower extremities can increase the suffering of patients, prolong hospitalization time, and increase medical expenses[8]. Once it falls off, it can easily cause pulmonary embolism, and severe cases can lead to death. The relevant guidelines[9, 10] point out that patients who have undergone major operations such as hip replacement without obvious bleeding tendency need to use anticoagulant drugs within 24 h after surgery, but there are still some patients who have DVT under the condition of standardized anticoagulant application. At present, there are many reports[11, 12] on DVT of the lower extremities after THA at home and abroad, but there are few studies on systemic analysis of DVT after artificial hip replacement, and the risk factors reported in related studies are relatively limited. It is necessary to further explore the related risk factors. Therefore, in this present study, we aimed to evaluate the influencing factors of DVT of lower limb after THA, to provide evidence support for the prevention and treatment of clinical THA.

## Methods

### Ethical issues

In this study, all methods were performed in accordance with the relevant guidelines and regulations. Our study had been verified and approved by the ethics committee of The Third Affiliated Hospital of Anhui Medical University (No.1,700,342), and all the included patients had signed the written informed consents.

### Patients

Patients who underwent THA in our hospital from January 1, 2017 to November 30, 2020 were selected as the research population. The inclusion criteria of patients in our study were: (1) The patient met the criteria for hip replacement and underwent the first THA in our hospital; (2) The age was ≥ 50 years old, regardless of gender; (3) Preoperative color Doppler ultrasound indicated that there was no DVT in both lower extremities during the examination; (4) The patient’s medical record was complete and available for data analysis. The exclusion criteria for patients were: (1) the pathological fractures caused by malignant tumors, tuberculosis which might influence the development of DVT; (2) DVT of the lower extremities existed before THA; (3) patients who disagreed to participate in this study.

### The diagnosis of DVT of lower extremity

The diagnosis of lower extremity DVT was based on the standards in the relevant diagnosis and treatment guidelines[13, 14]. Postoperative observation of the affected limbs for the following manifestations including limb swelling, pain, elevated skin temperature, skin color changes, venous return disorder, Homans sign, Neuhof’s sign. Color Doppler ultrasound indicated that there was no color blood flow signal and spectrum signal in the venous cavity, no collapse of the venous pressurized lumen, extremely low echo in the venous lumen, and irregular pulse Doppler spectrum. The ultrasound was generally used in the surgical leg, and if there were abnormal symptoms in another leg, we would also detect it for DVT. The inspection area for ultrasound scanning was from inguinal ligament to the distal leg. Patients underwent ultrasound examination every two days after surgery. And if necessary, the venography was performed.

### DVT preventions

We routinely conducted the mechanical post-operative DVT preventions for all patients as previous report[15]. We would encourage patients get out of bed as early as possible, and to massage the lower limb frequently and observe the status regularly. Besides, all the patients accepted the electrical stimulation for four days with Deming X100 massager(Deming biotechnology company, Wuxi, China), the massage started within 12 h after operation. The frequency was 30/50/30, the pulse width was 400/360/410, the massage continued 30 min each time. Besides, the intensity of stimulation was given differently based on the tolerance of patient, and it was set to the maximum intensity that could be tolerated by patients without discomfort. Besides, 10 mg per day of Rivaroxaban and 0.4ml of Low-molecular weight heparin calcium was used for the prevention of DVT.

### Data collection

We reviewed patients’ medical records, and we collected the general patient information including gender, age, body mass index(BMI), personal history, comorbid diseases, surgical factors including prosthesis type, duration of surgery, anesthesia method, postoperative factors including postoperative days in bed, anticoagulant drug application and laboratory indicators including preoperative D-dimer. Then we observed and analyzed the correlation between the above data and DVT.

### Data analysis

The observed data are statistically analyzed using SPSS23.0 statistical software. The measurement data are all expressed as mean ± standard deviation or percentage. According to the difference in the nature of each data, chi-square test, t-test were applied to identify the difference of DVT and no-DVT group. And logistic regression analyses were conducted to identify the potential risk factors of DVT in patients with THA. *P* < 0.05 indicated that the difference was statistically significant.

## Results

### The characteristics of included patients

A total of 182 THA patients were included, of whom 36 patients had suffered from DVT of lower limb, the incidence of DVT of lower limbs in patients with THA was 19.78 %. Of the 36 cases of DVT, there were 15 cases of proximal DVTs and 21 cases of distal DVTs. As presented in Table 1, there were significant differences in the age, BMI, diabetes, number of replacement, duration of surgery, type of prosthesis and duration of days in bed between DVT and no-DVT patients(all *P* < 0.05). And there were no significant differences in the gender, hypertension, hyperlipidemia, preoperative D-dimer, type of anesthesia and anticoagulant drugs use (all *P* > 0.05).

**Table 1:** The characteristics of included patients

Variables	DVT group(*n* = 36)	Non-DVT group(*n* = 146)	χ^2^/t	P
Male/female	20/16	92/54	1.046	0.102
Age(y)	72.14 ± 8.02	68.11 ± 9.35	4.328	0.025
BMI(kg/m^2^)	28.26 ± 4.55	26.02 ± 3.29	3.144	0.032
Diabetes	19(52.78 %)	25(17.12 %)	1.118	0.009
Hypertension	21(58.33 %)	77(52.71 %)	1.207	0.068
Hyperlipidemia	10(27.78 %)	42(28.77 %)	1.084	0.106
Preoperative D-dimer (mg/L)	0.69 ± 0.11	0.66 ± 0.12	1.230	0.074
Number of replacement joints				
Unilateral	11(30.56 %)	38(26.03 %)	1.140	0.031
Bilateral	25(69.44 %)	108(73.97 %)		
Duration of surgery(min)	133.62 ± 30.18	102 ± 27.42	9.124	0.015
Type of Anesthesia				
General anesthesia	16(44.44 %)	68(46.57 %)	1.122	0.078
Epidural anesthesia	20(55.56 %)	78(53.42 %)		
Type of prosthesis				
Biological prosthesis	6(16.67 %)	72(49.32 %)	1.205	0.036
Cemented prosthesis	30(83.33 %)	74(50.68 %)		
Duration of days in bed	4.24 ± 1.22	2.79 ± 1.02	1.177	0.042
Anticoagulant drugs				
Rivaroxaban	22(61.11 %)	85(58.22 %)	1.145	0.103
Low-molecular weight heparin calcium	14(38.89 %)	61(41.78 %)		

### The risk factors of DVT in patients with THA

We included the factors that were found to be significant differences between two group into further logistic regression, and the variable assignments of logistic regression were showed in Table 2.

**Table 2:** The variable assignments of logistic regression

Factors	Variables	Assignment
VTE	Y	Yes = 1, no = 2
Age(y)	X_1_	≥ 70 = 1, < 70 = 2
BMI(kg/m^2^)	X_2_	≥ 28 = 1, < 28 = 2
Diabetes	X_3_	Yes = 1, No = 2
Number of replacement joints	X_4_	Bilateral = 1, unilateral = 2
Duration of surgery(min)	X_5_	≥ 120 = 1, < 120 = 2
Type of prosthesis	X_6_	Cemented prosthesis = 1, biological prosthesis = 2
Duration of days in bed	X_7_	≥ 3 = 1, < 3 = 2

As indicated in Table 3, The logistic regression analysis found that age > 70y(OR4.406, 95 %CI1.744 ~ 6.134), BMI ≥ 28(OR2.275, 95 %CI1.181 ~ 4.531), diabetes(OR3.949, 95 %CI1.284 ~ 5.279), bilateral joint replacements(OR2.272, 95 %CI1.402 ~ 4.423), duration of surgery ≥ 120 min(OR3.081, 95 %CI1.293 ~ 5.308), cemented prosthesis(OR2.435, 95 %CI1.104 ~ 4.315), and duration of days in bed > 3 days(OR1.566, 95 %CI1.182 ~ 1.994) were the risk factors of DVT of lower limb in patients with THA.

**Table 3:** The logistic regression analysis on the risk factors of DVT in patients with THA

Variables	β	S`x	OR	95 %CI	p
Age > 70y	0.133	0.227	4.406	1.744 ~ 6.134	0.016
BMI ≥ 28	0.119	0.230	2.275	1.181 ~ 4.531	0.023
Diabetes	0.103	0.151	3.949	1.284 ~ 5.279	0.007
Bilateral joint replacements	0.147	0.183	2.272	1.402 ~ 4.423	0.033
Duration of surgery ≥ 120 min	0.149	0.102	3.081	1.293 ~ 5.308	0.026
Cemented prosthesis	0.126	0.117	2.435	1.104 ~ 4.315	0.042
Duration of days in bed > 3 days	0.113	0.124	1.566	1.182 ~ 1.994	0.037

## Discussions

DVT is a disorder of venous return caused by abnormal blood coagulation in the deep veins of the lower extremities, which completely or incompletely obstruct the blood vessels[16]. It is a common complication after THA and an important cause of unexpected deaths during the perioperative period of such patients[17]. Therefore, DVT has attracted extensive attention from clinical medical workers. Studies[18–20] have shown that the incidence of DVT in patients treated with THA is 14.13-20.18 %. The results of this study indicate that the incidence of lower limb DVT in patients after THA is 19.78 %, which is lower than the previous report[21, 22]. It may be related to the differences in the conditions of included patients, surgical method and perioperative preventive anticoagulation treatment amongst different studies.

Endometrial injury, venous blood flow stasis and blood hypercoagulability are the three factors that induce DVT[23]. The subjects of this study are elderly patients 50 years and older. The elasticity of blood vessels are poor and elder patients are complicated by the physiological or organic changes of multiple organs, and the perioperative period of lower extremity joint mobility and the amount of activity are significantly reduced, so the risk of DVT is higher[24–26]. We have found that age > 70y, BMI ≥ 28, diabetes, bilateral joint replacements, duration of surgery ≥ 120 min, cemented prosthesis, and duration of days in bed > 3 days were the independent risk factors of DVT of lower limb in patients with THA. It is clinically necessary to carry out early prevention and intervention for these risk factors of DVT to reduce the occurrence of DVT.

With the increase of age, the elasticity of blood vessels in patients decreases, the blood vessel walls are easily damaged, and elderly patients are prone to have primary diseases such as diabetes, which increases the incidence of DVT[27]. The results of our study are consistent with the findings of previous studies. BMI is a factor that affects the occurrence of DVT after surgery, which has been confirmed by many studies[28–30]. The guidelines set BMI ≥ 30 kg/m^2^ as an influencing factor of DVT. WHO uses BMI ≥ 30 kg/m^2^ as the obesity standard[31], and the WHO Western Pacific Region and the International Working Group recommend that for the Asian population BMI > 25 kg/m^2^ is taken as obesity [32]. Experts from the Chinese Obesity Working Group considered that BMI ≥ 28 kg/m2 is more appropriate for obesity[33], this study used the standard of BMI ≥ 28 kg/m^2^, the results have showed that BMI ≥ 28 kg/m^2^ is a risk factor affecting the occurrence of DVT. Therefore, for patients with BMI > 28, DVT should be warned in advance.

Many studies[34, 35] have reported that diabetes is a risk factor for DVT. Orthopedic surgery patients are in a state of stress. Diabetic patients’ blood glucose changes more than that of non-diabetic patients[36]. A large number of cytokines are released in the body during surgery, which easily activates the endogenous and exogenous coagulation system and increases the risk of thrombosis[37]. The results of this study suggest that blood glucose control in patients with THA perioperative should be strengthened to reduce the incidence of DVT. For patients with cemented prostheses, intraoperative bone cement can cause the release of mononuclear cytokines and the deformation and separation of endothelial cells, further covering the endothelial surface with fibrinogen, which in turn activates the exogenous blood coagulation pathway, resulting in high blood coagulation state, thereby increasing the risk of DVT[38]. Elderly patients with hip replacement are weak and require bed rest. Slow blood flow in the lower limb can also increase the risk of DVT[39]. The results of this study show that the days in bed ≥ 3 days after surgery is a risk factor for DVT. Therefore, it should be as early as possible to perform functional exercises of the lower limb to promote blood return to the lower limb and reduce the risk of thrombosis.

The prevention and treatment of DVT is of great significance to the prognosis of patients. It is necessary for doctors and nurses to strengthen preoperative assessment. Before surgery, it is necessary to understand the patient’s past medical history, general condition and blood coagulation, strengthen the screening of high-risk patients with DVT, and actively correct the effects of anemia, hypertension, diabetes and other cardiovascular diseases[40]. During the operation, the doctor should be familiar with the anatomy and surgical techniques, and reduce the operation and anesthesia duration. And the operation should be gentle and meticulous, and reduce unnecessary tissue damage, especially the intraoperative blood vessel damage, so as not to damage the vascular intima and induce thrombosis[41]. After the operation, the affected limb should be raised in certain position, and functional exercise should be performed as early as possible to increase venous blood return. Besides, the patients should regulate mood, eat lightly, and maintain smooth stools to reduce the obstruction of lower limb venous return caused by forced defecation and increased abdominal pressure[42, 43].

Several limitations must be concerned in this present study. Firstly, it is worth noting that the sample size of cases selected in this study is small and our study is a single-center study. The results of the study should be treated with caution. Secondly, patient risk stratification is a valid initial approach to ensure better management of patients undergoing THA and to predict who can benefit from a pharmacological preventive strategy, we did not perform individual thromboembolic risk before surgery in this present study. Thirdly, hyperglycemia has been found to be associated with many postoperative complications, in our clinical practice, we would correct the hyperglycemia before surgery. However, we did not detect the glycated hemoglobin routinely, since our study is a retrospective design, we could not collect the most data on the glycated hemoglobin, therefore we could not include those indicators for analysis. Besides, other factors including infections, medications, cancer, trauma and smoking et al. play a significant role in the total amount of post-surgical complications, limited by data, we did not investigate those factors in this present study, more studies on the association of those factors and postoperative DVT are needed. Future studies with larger samples and multi-centers need to further explore the risk factors of DVT in patients undergoing THA, to provide reliable evidence to the prophylaxis of DVT.

## Conclusions

In summary, the occurrence of DVT after THA is associated with many factors. In clinical work, attention should be paid to identify the risk factors that induce DVT and actively intervening are needed. The heath care provider should take effective measures targeted on the risk factors in time, and to guide patients to early perform functional exercises to reduce the incidence of DVT, thereby increasing the efficacy of THA and improving the life quality of patients.

## Data Availability

All data generated or analyzed during this study are included in this published article.

## Abbreviations

DVT: deep vein thrombosis
THA: total hip arthroplasty
